# *ESR1*, *PGR*, *ERBB2*, and *MKi67* mRNA expression in postmenopausal women with hormone receptor-positive early breast cancer: results from ABCSG Trial 6

**DOI:** 10.1016/j.esmoop.2021.100228

**Published:** 2021-08-07

**Authors:** M. Filipits, M. Rudas, C.F. Singer, F. Fitzal, Z. Bago-Horvath, R. Greil, M. Balic, S.F. Lax, S. Halper, W. Hulla, N.C. Wu, X. Liu, J. Weidler, M. Bates, D. Hlauschek, M. Gnant, P. Dubsky

**Affiliations:** 1 Institute of Cancer Research, Department of Medicine I, Breast Health Center and Comprehensive Cancer Center, Medical University of Vienna, Vienna, Austria; 2 Department of Gynecology, Breast Health Center and Comprehensive Cancer Center, Medical University of Vienna, Vienna, Austria; 3 Department of General Surgery, Breast Health Center and Comprehensive Cancer Center, Medical University of Vienna, Vienna, Austria; 4 Department of Pathology, Breast Health Center and Comprehensive Cancer Center, Medical University of Vienna, Vienna, Austria; 5 3^rd^ Medical Department, Salzburg Cancer Research Institute, Cancer Cluster Salzburg, Paracelsus Medical University Salzburg, Salzburg, Austria; 6 Department of Internal Medicine, Division of Oncology, Medical University Graz, Graz, Austria; 7 Department of Pathology, Hospital Graz II, Graz, Austria; 8 Johannes Kepler University Linz, Linz, Austria; 9 Department of Surgery, Breast Health Center, Hospital Wiener Neustadt, Wiener Neustadt, Austria; 10 Department of Clinical Pathology and Molecular Pathology, Breast Health Center, Hospital Wiener Neustadt, Wiener Neustadt, Austria; 11 Oncology Research and Development, Cepheid, Sunnyvale, USA; 12 Clinical Data Management and Analytics, Cepheid, Sunnyvale, USA; 13 Medical and Scientific Affairs and Strategy, Oncology, Cepheid, Sunnyvale, USA; 14 Austrian Breast and Colorectal Cancer Study Group (ABCSG), Vienna, Austria; 15 Comprehensive Cancer Center, Medical University of Vienna, Vienna, Austria; 16 St. Anna Breast Center, Hirslanden Klinik St. Anna, Lucerne, Switzerland; 17 Department of General Surgery, Comprehensive Cancer Center, Medical University of Vienna, Vienna, Austria

**Keywords:** early breast cancer, postmenopausal women, immunohistochemistry

## Abstract

**Background:**

The purpose of this study was to assess the concordance of real-time quantitative reverse transcription polymerase chain reaction (RT-qPCR) detection of *ESR1*, *PGR*, *ERBB2*, and *MKi67* messenger RNA (mRNA) in breast cancer tissues with central immunohistochemistry (IHC) in women treated within the prospective, randomized Austrian Breast and Colorectal Cancer Study Group (ABCSG) Trial 6.

**Patients and methods:**

We evaluated *ESR1*, *PGR*, *ERBB2*, and *MKi67* mRNA expression by Xpert® Breast Cancer STRAT4 (enables cartridge-based RT-qPCR detection of mRNA in formalin-fixed paraffin-embedded tissues) and estrogen receptor (ER), progesterone receptor (PR), human epidermal growth factor receptor 2 (HER2), and Ki67 protein expression by IHC [*in situ* hybridization (ISH) for HER2 IHC 2+] in 1115 surgical formalin-fixed paraffin-embedded specimens from patients of ABCSG Trial 6. Overall percent agreement (concordance), positive percent agreement (sensitivity), and negative percent agreement (specificity) between STRAT4 and IHC were determined for each marker. The primary objective of the study was concordance between STRAT4 mRNA measurements of *ESR1*, *PGR*, *ERBB2*, and *MKi67* with central reference laboratory IHC (and ISH for HER2 IHC 2+ cases). Time to distant recurrence was analyzed by Cox models.

**Results:**

All performance targets for ER, PR, and Ki67 were met. For HER2, the negative percent agreement target but not the positive percent agreement target was met. Concordance between STRAT4 and IHC was 98.9% for ER, 89.9% for PR, 98.2% for HER2, and 84.8% for Ki67 (excluding intermediate IHC 10%-20% staining). In univariable and multivariable Cox regression analyses, all four biomarkers tested by either STRAT4 RT-qPCR or by central IHC (ISH) had a comparable time to distant recurrence indicating similar prognostic value.

**Conclusions:**

With the exception of HER2, we demonstrate high concordance between centrally assessed IHC and mRNA measurements of ER, PR, and Ki67 as well as a high correlation of the two methods with clinical outcome. Thus, mRNA-based assessment by STRAT4 is a promising new tool for diagnostic and therapeutic decisions in breast cancer.

## Highlights

• Immunohistochemistry is the clinical gold standard for assessing ER, PR, HER2, and Ki67 expression in breast cancer.
• RNA expression assays provide an alternative approach to measuring these biomarkers.
• We demonstrate high concordance of STRAT4 mRNA detection in comparison to central immunohistochemistry.
• The STRAT4 assay is a promising new tool which can be carried out in <2 h and with lower costs.
• STRAT4 may be a cost-effective alternative to obtain standardized diagnostic results for breast cancer patients.

## Introduction

In early breast cancer, therapeutic decisions are based on a number of clinical and pathological factors, including the expression of biomarkers with established clinical utility and validity such as estrogen receptor (ER), progesterone receptor (PR), human epidermal growth factor receptor 2 (HER2), and a cellular marker for proliferation (Ki67). These four biomarkers have profound prognostic and therapeutic implications and are sometimes complemented by selected multigene assays.^1^^,^^2^

Semiquantitative assessment of the expression of ER, PR, HER2, and Ki67 proteins by immunohistochemistry (IHC) is currently the standard of care in routine clinical practice.^3^, ^4^, ^5^ In addition, in HER2 IHC 2+ (equivocal) cases, the status of *ERBB2* copy number is determined by *in situ* hybridization (ISH).^4^ The quality and accuracy of the determination is crucial for the optimal treatment of the patients. However, the intra- and inter-observer variation of the IHC of the four biomarkers, particularly Ki67, may be problematic.^4^, ^5^, ^6^ Tests measuring messenger RNA (mRNA) expression offer an alternative approach to assess these biomarkers but are not commonly used as a basis for selection of therapeutic regimens, and are currently not included in the current European Society for Medical Oncology (ESMO) or American Society of Clinical Oncology (ASCO)/College of American Pathologists (CAP) guidelines.^4^, ^5^, ^6^, ^7^, ^8^

The Xpert® Breast Cancer STRAT4 Assay (STRAT4) is a CE-IVD labeled *in vitro* diagnostic medical device which is not available in all countries and not available in the United States. STRAT4 is a cartridge-based test carried out on the GeneXpert® platform which automates nucleic acid extraction and purification and real-time quantitative reverse transcription polymerase chain reaction (RT-qPCR) detection of target genes [*ESR1* (NCBI Entrez Gene ID: 2099), *PGR* (NCBI Entrez Gene ID: 5241), *ERBB2* (NCBI Entrez Gene ID: 2064), and *MKi67* (NCBI Entrez Gene ID: 4288)] and a control gene (*CYFIP1*) mRNA in formalin-fixed paraffin-embedded (FFPE) tissues in <2 h.^7^ This system uses single-use disposable cartridges that hold the on-board PCR reagents and host the nucleic acid extraction, purification, and PCR process. The robustness and accuracy of the mRNA analysis platform strongly suggests that it may be equivalent to IHC but at a lower cost and in less time.

In this study, we investigated whether the STRAT4 assay is equivalent to centrally assessed IHC and ISH. To pursue this, we selected patients who were enrolled into the Austrian Breast and Colorectal Cancer Study Group Trial 6 (ABCSG Trial 6), a prospective randomized clinical trial comparing the efficacy of two endocrine treatment regimens with long-term follow-up.^9^

## Patients and methods

### ABCSG Trial 6

The objective of ABCSG Trial 6 was to compare the efficacy of adjuvant tamoxifen plus aminoglutethimide with tamoxifen alone. From December 1990 to December 1995, a total of 2020 patients were enrolled, of whom 1986 were assessed.^9^ Postmenopausal women with hormone receptor-positive breast cancer were randomized to receive either tamoxifen in combination with aminoglutethimide (500 mg/day) for the first 2 years followed by tamoxifen alone for 3 years or tamoxifen alone for 5 years.^9^ Tamoxifen was administered at 40 mg/day for the first 2 years and at 20 mg/day for 3 years. Since HER2 testing was not routinely carried out at the time of the trial execution, patients did not receive any anti-HER2 treatment. Furthermore, no adjuvant chemotherapy was administered. In the ABCSG Trial 6, aminoglutethimide given for 2 years in addition to tamoxifen for 5 years did not improve disease-free survival of postmenopausal patients with hormone receptor-positive, early-stage breast cancer.^9^ The maximum follow-up time for this study is 16 years, with a median of 11 years. The study was conducted in accordance with the Declaration of Helsinki, approved by the responsible ethics committees, and all patients gave written informed consent.

### Specimen collection and IHC

All patients (*n* = 2020) included in ABCSG Trial 6 were eligible for the translational study and participating centers were requested to provide a tumor block of their patients. Tumor specimens were obtained at the time of surgery before the adjuvant therapy. Details with regard to the collection of the samples and the preparation of sections were previously published as part of translational studies in this population.^10^^,^^11^ FFPE surgical breast cancer specimens from 1115 patients were retrospectively collected and used for IHC and RT-qPCR. A flowchart illustrating patient and sample selection is shown in Supplementary Figure S1, available at https://doi.org/10.1016/j.esmoop.2021.100228.

One hematoxylin–eosin-stained (H&E) slide was prepared from each paraffin block and reviewed by an experienced breast pathologist (MR) to confirm the presence of invasive breast carcinoma. Adjacent unstained 4 μm FFPE tissue sections were prepared for ER (SP1), PR (1E2), HER2 (4B5), and Ki67 (30-9) IHC on a BenchMark ULTRA® system (Ventana Medical Systems, Tucson, AZ) and Xpert Breast Cancer STRAT4 (CE-IVD) (Cepheid, Sunnyvale, CA) for *ESR1*, *PGR*, *ERBB2*, and *MKi67* mRNA testing. Additional tissue sections were used for HER2 ISH (INFORM HER2 dual probe) on a BenchMark ULTRA® system (Ventana) in cases with HER2 IHC 2+ results, as well as for secondary analyses using Dako antibodies for ER (EP1), PR (clone 1294), HER2 (RUO, polyclonal rabbit anti-human c-erbB2), and Ki67(MIB-1) on a Dako Omnis® (Agilent Technologies, Santa Clara, CA) system in selected cases. All IHC testing was centrally carried out in an academic reference laboratory at the Medical University of Vienna and evaluated according to current ESMO and ASCO/CAP guidelines by an experienced breast pathologist (MR) who was blinded for the clinical outcome.^4^, ^5^, ^6^

All invasive tumor cells on each slide were evaluated by visual estimation and interpretation of the results was limited to the invasive part of the tumor. For ER, PR, and Ki67, only nuclear staining was scored as positive, regardless of staining intensity. Results were documented as the percentage of ER-, PR-, and Ki67-stained nuclei, normalized to 1%, 5%, and then to 10% values. The positive cut-off was ≥1% for ER and PR regardless of the staining intensity. The results for Ki67 were grouped as follows: ≤5% (low), 10%-20% (intermediate), and ≥30% (high). HER2 IHC 3+ was considered positive and HER2 IHC 2+ cases underwent ISH analysis and were considered positive according to the current ASCO/CAP guidelines if the ratio was ≥2.0 or (after recount) the average *ERBB2* copy number was ≥6.0 per cell.^4^

### Xpert® breast cancer STRAT4 assay

The STRAT4 assay was developed for the simultaneous detection of *ESR1*, *PGR*, *ERBB2*, and *MKi67* mRNA relative to the expression level of a control gene, *CYFIP1*. The STRAT4 test cartridge enables nucleic acid purification, amplification, and real-time detection and quantification by PCR of mRNA targets by using a fully automated and completely integrated system after lysate sample preparation.

Briefly, for preparation of the lysate, one complete unstained FFPE section with tumor tissue cut at 10 μm was placed at the bottom of a 1.5 ml tube. If the FFPE section contained <30% invasive tumor, macrodissection was carried out and tumor areas were scraped off the slide. In addition, in cases with extensive intraductal component, ductal carcinoma *in situ* (DCIS) was removed before carrying out the assay.

After adding 1.2 ml of FFPE lysis buffer and 20 μl of proteinase K (Xpert® FFPE lysis kit, CE-IVD), the sample was mixed with a vortex mixer at maximum setting continuously for 10 s and incubated at 80°C for 30 min. After being vortexed for 5 s and briefly spun-down for 3 s, the sample was transferred to a 5 ml sample container and 1.2 ml of 95% ethanol was added. For each sample, 520 μl of the lysate was transferred to the GX STRAT4 cartridge. The filled cartridge was placed in the GX instrument and the assay was started. For cases with invalid *CYFIP1* [cycle threshold (Ct) value >35], a more concentrated tissue lysate was prepared according to the concentrated lysate sample preparation instructions per the manufacturer Xpert® FFPE lysis kit package insert instructions.

The entire assay, including off-board sample preparation, takes <2 h. The cartridge test results were reported as ΔCt measurements (ΔCt = *CYFIP1* Ct – target gene Ct). The pre-specified ΔCt thresholds for overexpression (positive results) relative to *CYFIP1* were ≥−1.0 for *ESR1*, ≥−3.5 for *PGR*, ≥−1.0 for *ERBB2*, and ≥−4.0 for *MKi67.* Any *CYFIP1* Ct value >35 is considered to have inadequate tumor cells to generate valid results for the primary analyses. The *CYFIP1* Ct cut-off for *ESR1* and *ERBB2* was set to ≤35 and for *PGR* and/or *MKi67* ΔCt values below the pre-specified positivity cut-offs to ≤31. Samples were classified as *PGR* or *MKi67* indeterminate if the *PGR* or *MKi67* ΔCt values were below the pre-specified ΔCt positivity cut-offs and the *CYFIP1* Ct value was >31 but ≤35.

### Statistical analysis

All data analyses were carried out according to a predefined statistical analysis plan.

The primary objective of the study was concordance between STRAT4 mRNA measurements of *ESR1*, *PGR*, *ERBB2*, and *MKi67* with central reference laboratory IHC (and ISH for HER2 IHC 2+ cases). For the concordance analysis, 2 × 2 tables were generated for each marker versus IHC/ISH as the reference and 95% confidence intervals (CIs) were generated using the Wilson score method to estimate the sensitivity and specificity for each marker. The predefined delta Ct values (relative to control Ct values) of each marker were used to define the positive/negative call for each category of the breast cancer subtypes. Overall percent agreement (OPA), positive percent agreement (PPA) (sensitivity), negative percent agreement (NPA) (specificity), and Kappa statistic between STRAT4 and IHC (IHC/ISH for HER2) were determined for each marker. The performance targets of this study were met if the lower 95% two-sided confidence limit for PPA and NPA exceeded the prospectively defined thresholds for each of the four targets: ER (PPA ≥80%, NPA ≥80%), PR (PPA ≥70%, NPA ≥65%), HER2 (PPA ≥75%, NPA ≥80%), Ki67 (PPA ≥65%, NPA ≥65%).

The secondary objective was the correlation of STRAT4 measurements with clinical outcomes of the patients. The secondary endpoint was time to distant recurrence (DR), defined as time from random assignment to occurrence of distant metastases. All secondary carcinoma (including contralateral breast cancer) and death of any cause were censored. In case of no distant metastases, secondary carcinoma, or death, the patient was censored at her last contact date. Time to DR was analyzed by Cox models and described by hazard ratios (HR with 95% CIs) and by Kaplan–Meier survival curves.

Categorical baseline data according to treatment arms were compared in univariable analysis using the chi-square or Fisher’s exact test depending on the expected cell frequencies. Continuous baseline data were compared using Wilcoxon test. All reported *P* values are results of two-sided tests. All results with *P* values <0.05 were considered statistically significant. Statistical analyses were carried out by members of the biostatistics group at ABCSG using statistical analysis system (SAS) software [SAS® version 9.3 (SAS Institute, Cary, NC) or higher]. This study meets the Reporting Recommendations for Tumor Marker Prognostic Studies (REMARK).

## Results

### Patient characteristics

Paraffin blocks with sufficient tumor quality were available from 1115 of the 2020 patients who were included in ABCSG Trial 6 and in whom long-term outcome data were available. Main clinical and laboratory parameters of all patients included in the present study compared with the patients without tumor blocks are summarized in Table 1. These 1115 patients were similar in age, tumor size, nodal status, tumor grade, and type of surgery compared with the 905 patients without available tumor blocks, but ER and PR expression of their tumors, and randomized treatment arm differed statistically significant between the two groups. Moreover, the time to DR observed in the study cohort was similar to the group of patients without tumor blocks (DR in patients without tumor blocks, 122 events; DR study cohort, 157 events; HR 0.95, 95% CI 0.75-1.20, *P* = 0.69). Thus, the patient cohort with tumor blocks available is representative of the whole trial population. STRAT4 and central IHC_Ventana_ results for all markers in all 1115 patients of the study cohort are shown in Table 2.

**Table 1:** Translational study cohort

	Patients with blocks (*n* = 1115)	Patients without blocks (*n* = 905)	ABCSG Trial 6 (*n* = 2020)	*P* value
Age (years)				0.25
Median (Q1-Q3)	64.8 (57.8-70.7)	64.2 (58.1-69.9)	64.6 (58.0-70.3)	
Min-Max	40.9-80.7	44.2-80.5	40.9-80.7	
Age (years), *n* (%)				0.12
≤50	30 (2.7)	21 (2.3)	51 (2.5)	
51-60	332 (29.8)	270 (29.8)	602 (29.8)	
61-70	430 (38.6)	389 (43.0)	819 (40.5)	
≥70	323 (29.0)	225 (24.9)	548 (27.1)	
Tumor size, *n* (%)				0.76
pT1	653 (58.6)	517 (57.1)	1170 (57.9)	
pT2	428 (38.4)	354 (39.1)	782 (38.7)	
pT3	30 (2.7)	29 (3.2)	59 (2.9)	
Missing	4 (0.4)	5 (0.6)	9 (0.4)	
Nodal status, *n* (%)				0.81
0 nodes	691 (62.0)	553 (61.1)	1244 (61.6)	
1-3 nodes	293 (26.3)	230 (25.4)	523 (25.9)	
4-10 nodes	95 (8.5)	87 (9.6)	182 (9.0)	
>10 nodes	32 (2.9)	30 (3.3)	62 (3.1)	
Missing	4 (0.4)	5 (0.6)	9 (0.4)	
Tumor grade local, *n* (%)				0.07
G1, G2, GX	888 (79.6)	683 (75.5)	1571 (77.8)	
G3	223 (20.0)	217 (24.0)	440 (21.8)	
Missing	4 (0.4)	5 (0.6)	9 (0.4)	
Estrogen receptor local, *n* (%)				<0.0001
Negative	17 (1.5)	37 (4.1)	54 (2.7)	
Positive	1081 (97.0)	814 (89.9)	1895 (93.8)	
Missing	17 (1.5)	54 (6.0)	71 (3.5)	
Progesterone receptor local, *n* (%)				<0.0001
Negative	239 (21.4)	161 (17.8)	400 (19.8)	
Positive	855 (76.7)	690 (76.2)	1545 (76.5)	
Missing	21 (1.9)	54 (6.0)	75 (3.7)	
Type of surgery, *n* (%)				0.50
Breast conserving	617 (55.3)	479 (52.9)	1096 (54.3)	
Mastectomy	494 (44.3)	421 (46.5)	915 (45.3)	
Missing	4 (0.4)	5 (0.6)	9 (0.4)	
Treatment arm, *n* (%)				0.003
Tamoxifen	590 (52.9)	418 (46.2)	1008 (49.9)	
Tamoxifen + aminoglutethimide	525 (47.1)	487 (53.8)	1012 (50.1)	

ABCSG, Austrian Breast and Colorectal Cancer Study Group.

**Table 2:** STRAT4 and central IHC_Ventana+ISH_ results in ABCSG Trial 6

	Tamoxifen (*n* = 590)	Tamoxifen + aminoglutethimide (*n* = 525)	ABCSG Trial 6 (*n* = 1115)	*P* value
*ESR1*, *n* (%)				0.52
Positive	553 (93.7)	492 (93.7)	1045 (93.7)	
Negative	14 (2.4)	17 (3.2)	31 (2.8)	
Invalid	23 (3.9)	16 (3.0)	39 (3.5)	
*PGR*, *n* (%)				0.08
Positive	429 (72.7)	416 (79.2)	845 (75.8)	
Negative	105 (17.8)	73 (13.9)	178 (16.0)	
Invalid	23 (3.9)	16 (3.0)	39 (3.5)	
Indeterminate	33 (5.6)	20 (3.8)	53 (4.8)	
*ERBB2*, *n* (%)				0.32
Positive	18 (3.1)	24 (4.6)	42 (3.8)	
Negative	549 (93.1)	485 (92.4)	1034 (92.7)	
Invalid	23 (3.9)	16 (3.0)	39 (3.5)	
*MKi67*, *n* (%)				0.12
Positive	263 (44.6)	245 (46.7)	508 (45.6)	
Negative	225 (38.1)	216 (41.1)	441 (39.6)	
Invalid	23 (3.9)	16 (3.0)	39 (3.5)	
Indeterminate	79 (13.4)	48 (9.1)	127 (11.4)	
ER_Ventana_, *n* (%)				0.78
Positive	579 (98.1)	514 (97.9)	1093 (98.0)	
Negative	11 (1.9)	11 (2.1)	22 (2.0)	
PR_Ventana_, *n* (%)				0.18
Positive	508 (86.1)	466 (88.8)	974 (87.4)	
Negative	82 (13.9)	59 (11.2)	141 (12.6)	
HER2_Ventana+ISH_, *n* (%)				0.17
Positive	27 (4.6)	32 (6.1)	59 (5.3)	
Negative	563 (95.4)	491 (93.5)	1054 (94.5)	
Missing	0	2 (0.4)	2 (0.2)	
Ki67_Ventana_, *n* (%)				<0.0001
≤5%	263 (44.6)	165 (31.4)	428 (38.4)	
10%-20%	261 (44.2)	252 (48.0)	513 (46.0)	
≥30%	66 (11.2)	108 (20.6)	174 (15.6)	

ABCSG, Austrian Breast and Colorectal Cancer Study Group; ER, estrogen receptor; HER2, human epidermal growth factor receptor 2; IHC, immunohistochemistry; ISH, *in situ* hybridization; PR, progesterone receptor.

### Concordance between STRAT4 ESR1 mRNA and ER immunoreactivity

**Figure 1:**
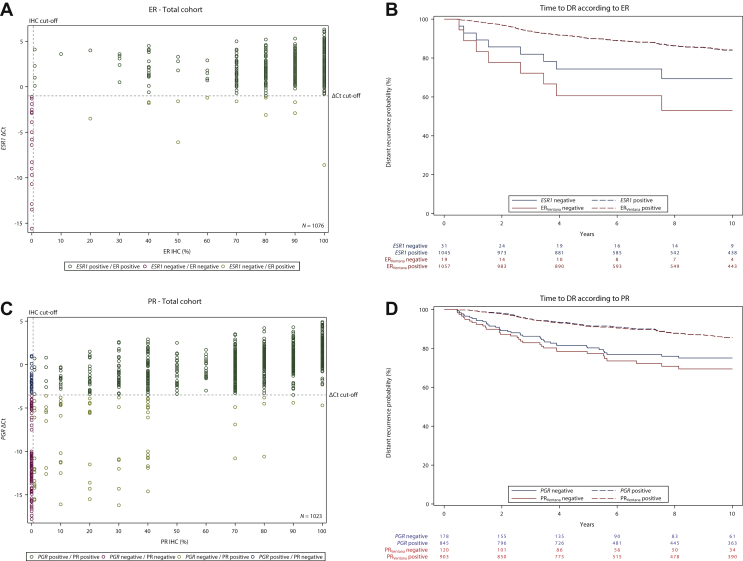

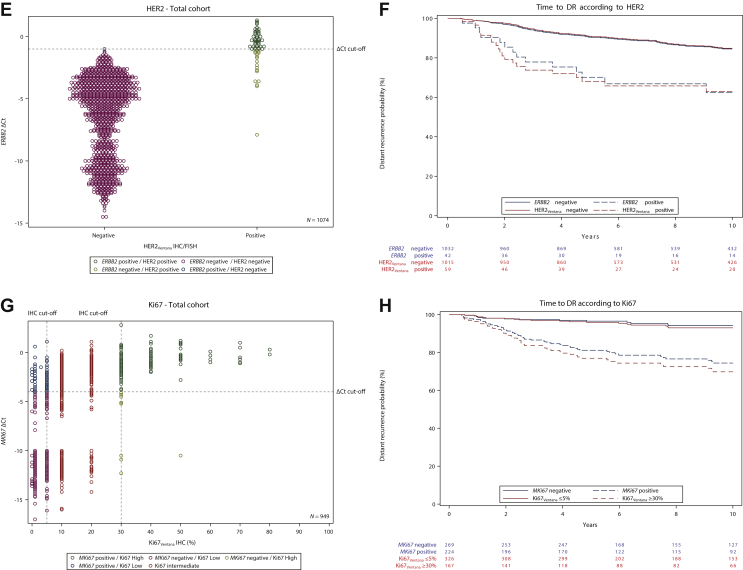
Scatter plots of STRAT4 messenger RNA results and central IHC_Ventana_ (A, C, E, G) and Kaplan–Meier plots for time to DR according to *ESR1*/ER_Ventana_ (B), *PGR*/PR_Ventana_ (D), *ERBB2*/HER2_Ventana+ISH_ (F), *MKi67*/Ki67_Ventana_ (H) expression in the total ABCSG Trial 6 study cohort. ABCSG, Austrian Breast and Colorectal Cancer Study Group; Ct, cycle threshold; DR, distant recurrence; ER, estrogen receptor; FISH, fluorescence *in situ* hybridization; HER2, human epidermal growth factor receptor 2; IHC, immunohistochemistry; ISH, *in situ* hybridization; PR, progesterone receptor.

The primary objective for ER was met. The 39 (3.5%) cases with invalid STRAT4 *ESR1* results were excluded from the concordance analysis (Table 2). The lower limits of the 95% CIs for PPA and NPA between STRAT4 *ESR1* mRNA measurements and ER protein results determined by central IHC_Ventana_ in 1076 cases were 98.0% and 87.5%, respectively (concordance 98.9%) (Table 3). *ESR1* ΔCt values were plotted against percent positive ER staining treated as continuous variables and for the same samples. These data show high concordance between STRAT4 *ESR1* mRNA and central ER IHC_Ventana_ (Figure 1A).

**Table 3:** Concordance between IHC_Ventana_ versus STRAT4 and IHC_Ventana_ versus IHC_Dako_

Total study population								
Marker	IHC_Ventana+_/STRAT4+	IHC_Ventana−_/STRAT4+	IHC_Ventana+_/STRAT4−	IHC_Ventana−_/STRAT4−	Concordance OPA, % (95% CI)	Sensitivity PPA, % (95% CI)	Specificity NPA, % (95% CI)	Kappa statistic (95% CI)
*ESR1*/ER	1045	0	12	19	98.9 (98.1% to 99.4%)	98.9 (98.0% to 99.3%)	100.0 (87.5% to 100.0%)	0.76 (0.62% to 0.89%)
*PGR*/PR	822	23	81	97	89.8 (87.8% to 91.5%)	91.0 (89.0% to 92.7%)	80.8 (72.9% to 86.9%)	0.59 (0.53% to 0.66%)
*ERBB2*/HER2	41	1	18	1014	98.2 (97.3% to 98.9%)	69.5 (56.9% to 79.7%)	99.9 (99.4% to 100.0%)	0.80 (0.72% to 0.89%)
*MKi67*/Ki67	158	66	9	260	84.8 (81.3% to 87.7%)	94.6 (90.1% to 97.1%)	79.8 (75.1% to 83.8%)	0.69 (0.62% to 0.75%)

Tamoxifen plus aminoglutethimide arm								
Marker	IHC_Ventana+_/IHC_Dako+_	IHC_Ventana−_/IHC_Dako+_	IHC_Ventana+_/IHC_Dako−_	IHC_Ventana−_/IHC_Dako−_	Concordance OPA, % (95% CI)	Sensitivity PPA, % (95% CI)	Specificity NPA, % (95% CI)	Kappa statistic (95% CI)
ER	508	0	6	11	98.9 (97.5% to 99.5%)	96.6 (97.5% to 99.5%)	100.0 (80.3% to 100.0%)	0.78 (0.61% to 0.95%)
PR	385	2	81	57	84.2 (80.8% to 87.1%)	82.6 (78.9% to 85.8%)	96.6 (88.5% to 99.1%)	0.50 (0.41% to 0.59%)
HER2	32	7	0	466	98.6 (97.2% to 99.3%)	100.0 (92.2% to 100.0%)	98.5 (97.0% to 99.3%)	0.89 (0.82% to 0.97%)
Ki67	48	0	16	155	92.7 (88.5% to 95.5%)	75.0 (63.2% to 84.0%)	100.0 (98.3% to 100.0%)	0.81 (0.72% to 0.90%)

CI, confidence interval; ER, estrogen receptor; HER2, human epidermal growth factor receptor 2; IHC, immunohistochemistry; ISH, *in situ* hybridization; NPA, negative percent agreement; OPA, overall percent agreement; PPA, positive percent agreement; PR, progesterone receptor.

### Concordance between STRAT4 PGR mRNA and PR immunoreactivity

The primary objective for PR was met. The 53 (4.8%) cases with indeterminate and 39 (3.5%) cases with invalid STRAT4 *PGR* results were excluded from the concordance analysis (Table 2). The lower limits of the 95% CIs for PPA and NPA of the STRAT4 *PGR* mRNA results compared with PR protein results assessed by central IHC_Ventana_ in 1023 cases were 89.0% and 72.9%, respectively (concordance 89.8%) (Table 3). Scatter plots of STRAT4 *PGR* mRNA compared with PR protein suggest a positive correlation (Figure 1C).

### Concordance between ERBB2 mRNA and HER2 immunoreactivity

The primary objective for HER2 was not met for PPA but was met for NPA. The 39 (3.5%) cases with invalid STRAT4 *ERBB2* results and the two cases with missing HER2 IHC_Ventana_ results were excluded from the concordance analysis (Table 2). The lower limits of the 95% CIs for PPA and NPA between STRAT4 *ERBB2* mRNA and HER2 IHC_Ventana_ plus ISH, where IHC_Ventana_ 2+ samples were tested by ISH and categorized as either ISH-positive or ISH-negative, in 1074 cases were 56.9% and 99.4%, respectively (concordance 98.2%) (Table 3). A comparison of STRAT4 *ERBB2* ΔCt values and IHC_Ventana_ results classified as IHC_Ventana_ plus ISH-negative and IHC_Ventana_ plus ISH-positive cases is shown in a scatter plot (Figure 1E).

### Concordance between MKi67 mRNA and Ki67 labeling index

The primary objective for Ki67 was met. For *MKi67*/Ki67 concordance, 127 (11.4%) samples with indeterminate STRAT4 results, 39 (3.5%) samples with invalid STRAT4 *MKi67* results, and 513 samples with intermediate (10%-20%) Ki67 protein expression were excluded from the concordance analysis (Table 2). The lower limits of the 95% CIs for PPA and NPA between STRAT4 *MKi67* mRNA results and Ki67 protein expression by IHC_Ventana_ in 493 cases were 90.1% and 75.1%, respectively (concordance 84.8%) (Table 3). A scatter plot of *MKi67* ΔCt values with Ki67 IHC_Ventana_ results (*n* = 949) is shown in Figure 1G.

### Concordance between IHC_Ventana_ and IHC_Dako_

The concordance between two different IHC platforms—Ventana BenchMark Ultra® and Dako Omnis®—which are currently used in routine clinical practice to determine the protein expression of ER, PR, HER2, and Ki67, was assessed in 525 patients of the tamoxifen plus aminoglutethimide arm of ABCSG Trial 6. All IHC stainings were evaluated by one experienced breast pathologist. The OPA, PPA, and NPA of ER, PR, HER2, and Ki67 expression determined by IHC_Ventana_ compared with IHC_Dako_ are shown in Table 3 and demonstrate a very similar concordance between the two IHC platforms and STRAT4 mRNA measurements and central IHC.

### Time to DR analyses in the study cohort

At a median follow-up of 11 years, 157 of 1115 (14.1%) patients of the study population had developed distant metastases; 82 (13.9%) patients in the tamoxifen arm and 75 (14.3%) patients in the tamoxifen plus aminoglutethimide arm. A total of 937 patients with 138 DR events for whom all covariates were available were included in univariable and multivariable Cox models.

In univariable analyses, tumor size, nodal status, *ESR1* mRNA expression, *PGR* mRNA expression, *ERBB2* mRNA expression, *MKi67* mRNA expression, ER_Ventana_ expression central, PR_Ventana_ expression central, HER2_Ventana+ISH_ central, and Ki67_Ventana_ expression central were significantly associated with time to DR (Figure 1B, D, F, H; Supplementary Figure S2, available at https://doi.org/10.1016/j.esmoop.2021.100228 and Table 4).

**Table 4:** Cox proportional hazard models

	Univariable models		Multivariable model STRAT4		Multivariable model IHC_Ventana_	
	HR (95% CI)	*P* value∗	HR (95% CI)	*P* value∗	HR (95% CI)	*P* value∗
Age	0.98 (0.96-1.00)	0.12	0.98 (0.96-1.00)	0.08	0.98 (0.96-1.00)	0.11
Tumor size		<0.0001		0.007		0.003
pT2 versus pT1	2.34 (1.65-3.31)	<0.0001	1.80 (1.24-2.59)	0.002	1.86 (1.30-2.68)	0.0007
pT3 versus pT1	2.92 (1.26-6.79)	0.013	1.41 (0.58-3.41)	0.45	1.23 (0.50-3.06)	0.65
Nodal status		<0.0001		<0.0001		<0.0001
1-3 nodes versus 0 nodes	2.42 (1.62-3.60)	<0.0001	2.16 (1.44-3.24)	0.0002	2.08 (1.37-3.14)	0.0005
4-10 nodes versus 0 nodes	5.60 (3.55-8.83)	<0.0001	5.16 (3.20-8.33)	<0.0001	5.40 (3.34-8.73)	<0.0001
>10 nodes versus 0 nodes	6.36 (3.30-12.24)	<0.0001	4.70 (2.33-9.49)	<0.0001	4.23 (2.06-8.69)	<0.0001
Tumor grade (local)		0.41		0.44		0.47
G2 versus G1	1.44 (0.84-2.46)	0.18	0.85 (0.49-1.49)	0.57	0.91 (0.53-1.59)	0.75
G3 versus G1	1.66 (0.91-3.03)	0.10	0.64 (0.33-1.22)	0.17	0.67 (0.35-1.27)	0.22
GX versus G1	1.25 (0.53-2.92)	0.61	1.04 (0.44-2.45)	0.93	1.01 (0.42-2.41)	0.98
*ESR1*						
Positive versus negative	0.40 (0.20-0.82)	0.01	0.61 (0.28-1.34)	0.22		
*PGR*						
Positive versus negative	0.50 (0.34-0.71)	0.0002	0.58 (0.39-0.87)	0.008		
*ERBB2*						
Positive versus negative	2.92 (1.68-5.08)	0.0001	1.95 (1.09-3.50)	0.02		
*MKi67*						
Positive versus negative	2.35 (1.62-3.39)	<0.0001	1.94 (1.31-2.87)	0.0009		
ER_Ventana_						
Positive versus negative	0.28 (0.13-0.60)	0.001			0.49 (0.20-1.17)	0.11
PR_Ventana_						
Positive versus negative	0.41 (0.28-0.62)	<0.0001			0.53 (0.33-0.84)	0.007
HER2_Ventana_						
Positive versus negative	3.05 (1.88-4.96)	<0.0001			1.56 (0.91-2.68)	0.11
Ki67_Ventana_		<0.0001				0.0002
10%-20% versus <10%	2.56 (1.59-4.12)	0.0001			2.36 (1.46-3.82)	0.0005
>20% versus <10%	4.55 (2.72-7.61)	<0.0001			3.13 (1.78-5.51)	<0.0001
Ki67_Ventana_						
>20% versus ≤20%	2.42 (1.69-3.47)	<0.0001				

A total of 937 patients with 138 distant recurrence (DR) events for whom all covariates were available were included.

CI, confidence interval; ER, estrogen receptor; HER2, human epidermal growth factor receptor 2; HR, hazard ratio; IHC, immunohistochemistry; PR, progesterone receptor.

∗ Wald chi-square *P* value. For variables with 3+ categories the first *P* value shows the Type 3 test Wald chi-square *P* value.

We carried out two separate multivariable Cox models combining age, tumor size, nodal status, and tumor grade with either STRAT4 mRNA measurements or central IHC_Ventana_ results (Table 4). In multivariable analyses that included STRAT4 mRNA measurements, tumor size, nodal status, *PGR*, *ERBB2*, and *MKi67* were independent prognostic factors for time to DR (Table 4). In the second multivariable model which included central IHC_Ventana_ results, tumor size, nodal status, PR_Ventana_, and Ki67_Ventana_ were independently associated with time to DR (Table 4).

Finally, we compared the STRAT4 mRNA results for each marker with both central IHC_Ventana_ and central IHC_Dako_ with time to DR in the tamoxifen plus aminoglutethimide arm Supplementary Figure S3, available at https://doi.org/10.1016/j.esmoop.2021.100228). In these analyses, we found similar correlations for *ESR1*/ER, *PGR*/PR, *ERBB2*/HER2, and *MKi67*/Ki67 with time to DR.

## Discussion

IHC allows a visual estimation of protein expression in FFPE tissue sections from breast cancer samples. The distribution of the stained antigen can be assessed within tumor cells, stroma, and non-neoplastic tissue. Although IHC assessment follows a semiquantitative approach using an immunoreactive score, a simple final grouping as positive and negative is highly predictive of response both to anti-hormonal and anti-HER2 treatment. In addition, ER, PR, HER2, and Ki67 deliver important prognostic information, complimentary to clinicopathological parameters.^12^

However, testing for these four biomarkers by IHC in the daily practice has its obstacles and pitfalls. Several guidelines and recommendations acknowledge that up to 20% of worldwide ER/PR testing may be inaccurate due to (i) pre-analytic variability, (ii) variable implementation of thresholds, and (iii) differing criteria of test interpretation.^5^ A similar rate of false-negative and false-positive results has been described for HER2 testing.^4^ Finally, the clinical utility of Ki67 is limited, although measures of cellular proliferation are prognostically important and validation has been followed for more than a decade.^13^ Notably, for ER-positive tumors, a broad consensus has only been found for Ki67 values of ≤5% and ≥30%.^6^ Ki67 particularly exemplifies that whereas IHC is a gold standard in the assessment of biomarkers for breast cancer, each of the four biomarkers must be separately critically reviewed. This review should include its analytic and clinical validity and utility with respect to the full biomarker context and clinical setting. Particularly, for hormone receptor-positive, early breast cancer it needs to be emphasized that not a single biomarker, but the pattern of the four biomarkers and clinicopathological parameters including stage (reflecting tumor size and nodal status) and histopathological grade is informative for clinical decisions.^12^

In the first part of our study, we assessed the concordance of the STRAT4 assay to central IHC in 1115 cases from the ABCSG Trial 6 using archived FFPE breast cancer tissue and found a high overall agreement for all four biomarkers. *ESR1*/ER, possibly the marker with both the highest predictive and prognostic value in breast cancer, reached an OPA of 98.9%. The identical value was found when two IHC platforms (Ventana BenchMark Ultra® and Dako Omnis®) were compared in a subset of the cohort. Similar concordance (OPA 89.8%) was reached for *PGR*/PR. In ∼80 cases IHC was positive in >1% of cells, whereas the mRNA threshold for positivity was not reached. The descriptive assessment in the scatter plots allows the speculation that mRNA degradation may have occurred in these >20 years old samples. Despite this discordance, primary endpoints of the study were met.

The ABCSG Trial 6 cohort was randomized several years before HER2 assessment was introduced into breast cancer management. Given the endocrine therapy question behind the clinical trial, only 5.3% of the biomarker cohort showed HER2 positivity. Only 41 of the 59 HER2-positive/amplified samples crossed the ΔCt value of mRNA positivity. Due to the low pre-test likelihood in the cohort, the statistical analysis provides wide CIs for PPA and its predefined lower limit was not reached (56.5%), although the OPA was 98.2%. Given the low number of HER2-positive cases in the sample, a visual or statistical analysis of discordance is not informative and should be carried out in a more suitable cohort.

Similar to the recent consensus on the clinical utility of Ki67 in hormone receptor-positive breast cancer,^6^ the predefined analysis plan of our study omitted samples with Ki67 values between 10% and 20%. Since both the analytic and clinical utility of the marker within this value range is questionable (in ER-positive/HER2-negative, early breast cancer), the study focused on values ≤5% and ≥30%. Both the defined statistical targets (PPA/NPA) and OPA show good concordance between the two methods. Perhaps not surprisingly both concordance and discordance were similar if Ki67 expression was compared between the two IHC platforms. In summary, the biomarker assessment by IHC and by mRNA expression showed high concordance. In addition, we are able to demonstrate that a minor discordance can be shown by comparison of two IHC staining platforms. In particular, this understanding of IHC assessment as a ‘moving target’ confirms our rationale to investigate the prognostic value of markers in addition to the concordance of results.

The use of the ABCSG Trial 6 biomarker cohort allowed interrogation of well-monitored, prospectively assessed, long-term oncologic event data from a prospective phase III clinical trial. Thus, as a secondary endpoint to biomarker concordance, we were able to compare the prognostic association of the four mRNA biomarkers to IHC, which was centrally tested. To the best of our knowledge, we show for the first time (by retrospective analysis of a prospective phase III clinical dataset) a significant and comparable association of the four biomarkers assessed both by IHC and mRNA analysis to the occurrence of DR. In the univariable models of all markers (both IHC and mRNA assessment) all four markers show significant association with time to DR; the effect size of all four biomarkers is comparable between mRNA and protein expression. In multivariable analyses, both *ESR1* and ER did not show statistically independent association due to the almost uniformly *ESR1*/ER positive distribution of the cohort. In contrast, both *PGR*/PR and *MKi67*/Ki67 were independent prognostic factors; only *ERBB2* mRNA, but not HER2 IHC/ISH, showed significant and independent correlation with time to DR. Thus, the STRAT4 assay provides similar prognostic values as central assessment of IHC.

Given the positive study results, it is important to speculate on how the STRAT4 assay could be implemented into the pathology work flow. Currently, the gold standard for the assessment of ER, PR, HER2, and Ki67 is ICH on FFPE tumor tissue which is very well established within the pathology community. The STRAT4 assay is a promising new tool which can be carried out in <2 h and—in comparison with IHC plus HER2 ISH—with lower costs. Therefore, STRAT4 may be a cost-effective alternative to obtain standardized diagnostic results for breast cancer patients.

The STRAT4 assay has some limitations, however, particularly for invasive carcinoma with extensive intraductal component, DCIS with microinvasion, and minimal residual tumor after preoperative therapy. Furthermore, larger amounts of adjacent normal breast tissue as well as of other precursor lesions, such as atypical ductal hyperplasia or lobular neoplasia, need also to be removed before carrying out the assay on invasive carcinoma, particularly if the tumor is ER/PR-negative. The standard pathology procedure in pathology using H&E slides for histological diagnosis should easily allow the selection of invasive tumor areas for further mRNA analysis and the exclusion of non-neoplastic and non-invasive tumor tissue. Thus, a pathology work flow with proper quality assurance will guarantee adequate case selection for the mRNA assay.

As described in our own data, the STRAT4 assay can also deliver ‘borderline’ or indeterminate results which can be due to analytic problems or simply because the sample shows low or heterogeneous expression of the biomarker. In this case, especially if the biomarker result will lead to clear clinical consequences (e.g. ER-positive versus ER-negative) it is crucial to complement the assay with IHC.

Although this study was sufficiently powered to address the concordance and prognostic information of all four biomarkers, the distribution of biomarkers in ABCSG Trial 6 is not representative of all breast cancer cohorts, due to the inclusion/exclusion criteria of this trial mainly addressing an endocrine therapy question. Additional studies in chemotherapy-treated cohorts (with more ER-negative and HER2-positive samples) and in core biopsies are underway to gain better insights and confirmation of our results.

Previously, two studies determined mRNA expression levels of *ESR1*, *PGR*, *ERBB2*, and *MKi67* in FFPE tissue.^7^^,^^8^ One study reported high concordance between ER, PR, HER2, and Ki67 IHC/ISH assessment and mRNA measurements using the STRAT4 assay and showed similar results to the present study.^7^ The second study reported high inter-site and intra-site reproducibility of *ESR1*, *PGR*, *ERBB2*, and *MKi67* mRNA measurements with the MammaTyper® test and showed a precise and reproducible assessment of the four breast cancer biomarkers.^8^ Therefore, the currently available data show that mRNA-based measurement of key breast cancer markers shows promise.

In summary, this biomarker analysis shows, with the exception of HER2, high concordance between ER, PR, and Ki67 IHC protein assessment and the STRAT4 mRNA assay, in addition to confirming the prognostic value of each biomarker. Thus, mRNA-based assessment by STRAT4 is a promising new tool for cost-effective and standardized breast cancer diagnostic and therapeutic decisions.
