# Supplementary figures and images for: ESR1, PGR, ERBB2, and MKi67 mRNA expression in postmenopausal women with hormone receptor-positive early breast cancer: results from ABCSG Trial 6
Source: ESMO Open. 2021 Aug 7;6(4):100228. doi: 10.1016/j.esmoop.2021.100228 (PMC8358421; doi:10.1016/j.esmoop.2021.100228)

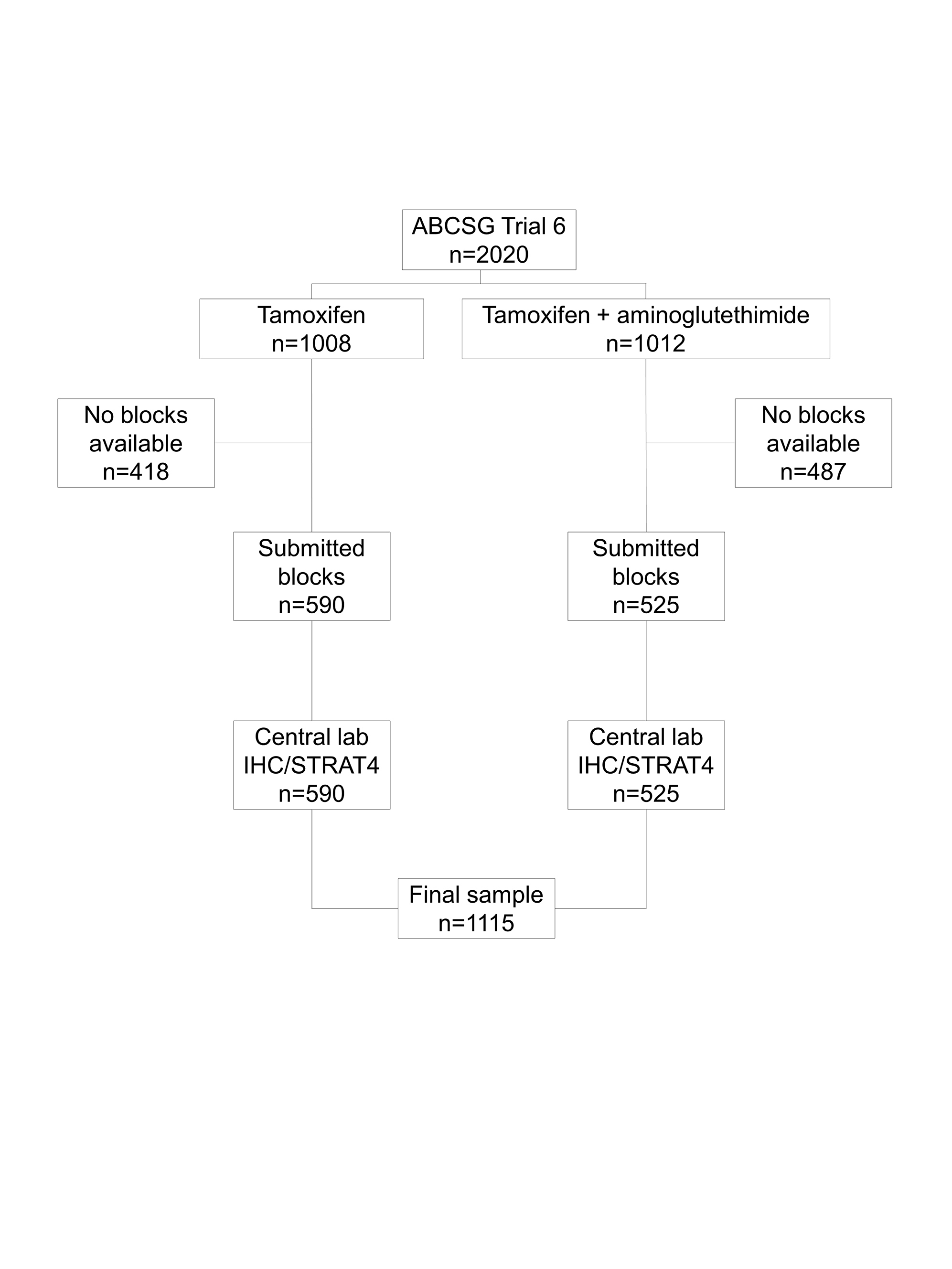

Supplement: Figure S1 [file figs1.jpg]

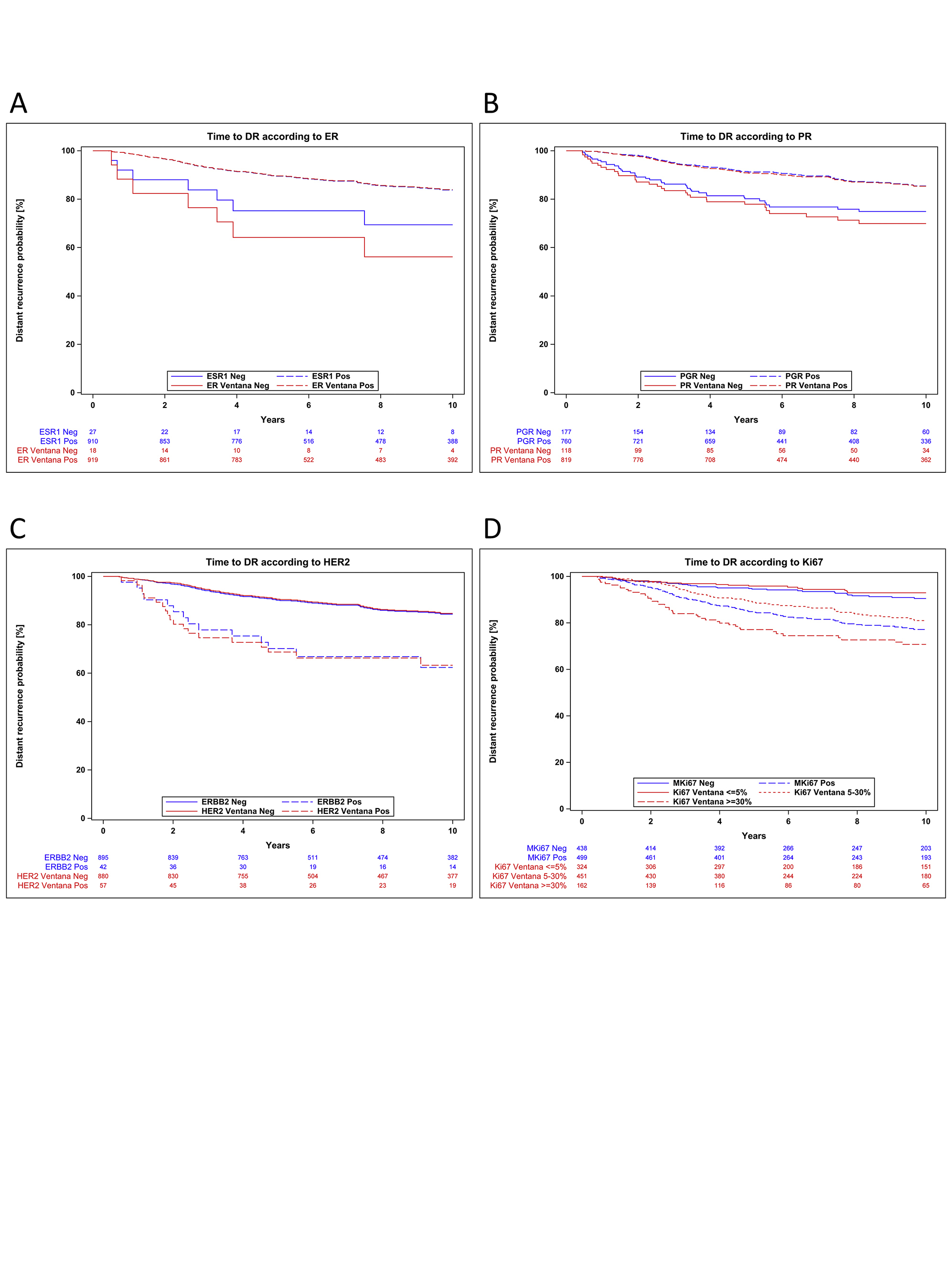

Supplement: Figure S2 [file figs2.jpg]

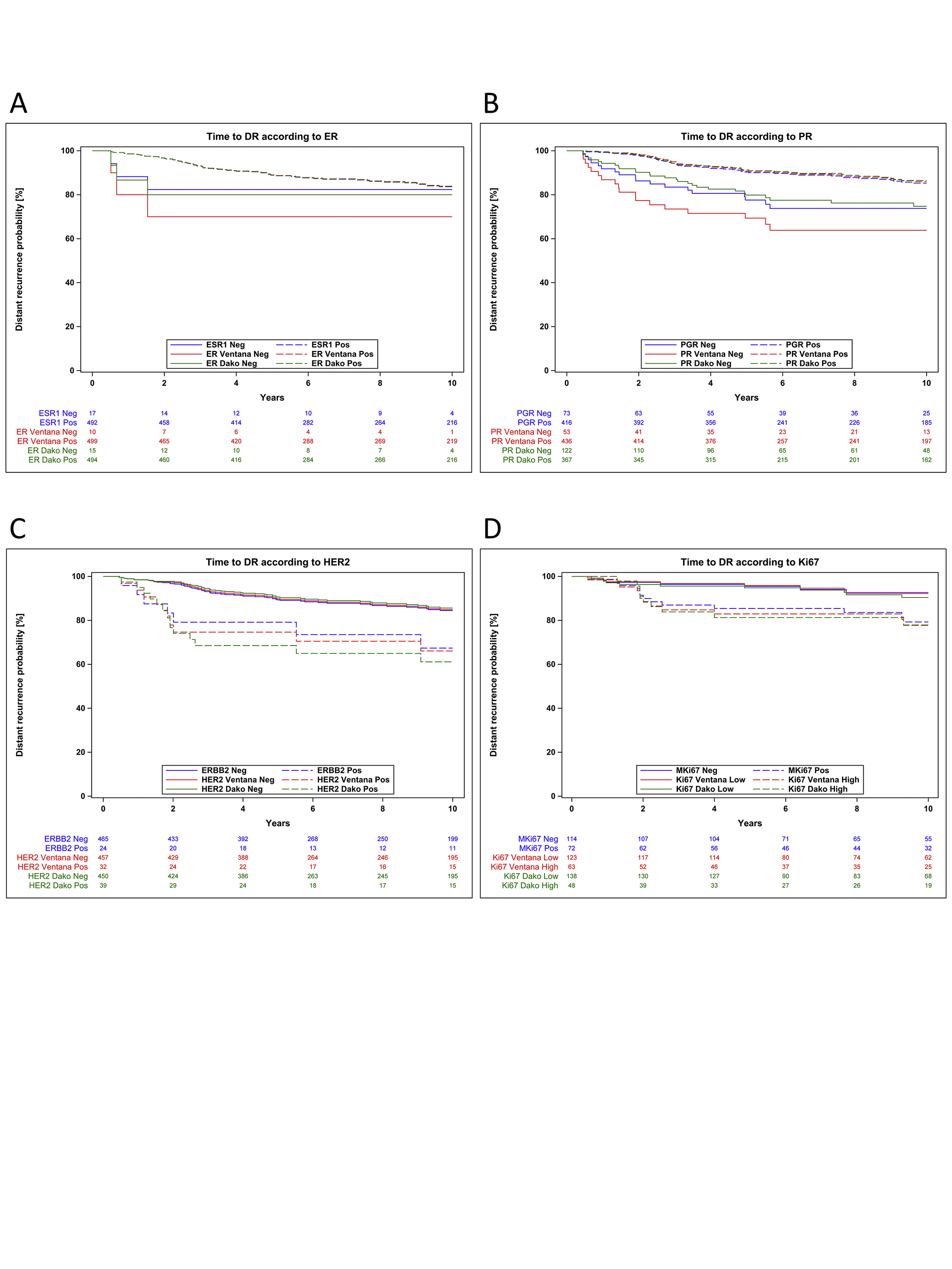

Supplement: Figure S3 [file figs3.jpg]
